# Using standard-length compactors to implant short humeral stems in total shoulder arthroplasty: A cadaver study of humeral stem alignment

**DOI:** 10.1371/journal.pone.0268075

**Published:** 2022-05-05

**Authors:** Stanislas Gunst, Ana Nigues, Jérôme Vogels, Elvire Servien, Sébastien Lustig, Laurent Nove-Josserand, Philippe Collotte

**Affiliations:** 1 Orthopaedics Surgery and Sports Medicine Department, FIFA Medical Center of Excellence, Croix-Rousse Hospital, Lyon University Hospital, Lyon, France; 2 Univ Lyon, Claude Bernard Lyon 1 University, IFSTTAR, LBMC UMR_T9406, Lyon, France; 3 Hand and Upper Extremity Surgical Institute, Clinique du Medipole, Villeurbanne, France; 4 LIBM–EA 7424, Interuniversity Laboratory of Biology of Mobility, Claude Bernard Lyon 1 University, Lyon, France; 5 Ramsay Générale de Santé, Jean Mermoz Private Hospital, Centre Orthopédique Santy, Lyon, France; Indiana University School of Medicine, UNITED STATES

## Abstract

**Background:**

Short-stem implants in shoulder arthroplasty were recently developed and reported clinical outcomes are good. However, radiological analysis often reveals humeral stem misalignment in the frontal plane, along with high filling ratios that can lead to proximal bone remodeling under stress shielding. The aim of this cadaveric study was to test whether using compactors for standard-length (> 100 mm) stems to implant short (< 100 mm) stems reduces the risk of stem misalignment without compromising in terms of a higher filling ratio.

**Methods:**

In a cadaveric study, twenty short stems were implanted using instrumentation for standard-length stems. Alignment and filling ratios were evaluated on anteroposterior radiographs for both the compactors and the stems. The angular deviations (*α*) from the humeral axis of the compactors and the short stems were measured. Misalignment was defined as |*α*| > 5°. Metaphyseal and diaphyseal filling ratios were calculated and defined as either high (≥ 0.7) or low (< 0.7).

**Results:**

The median angular deviations of the compactors and the short stems were respectively 1.6° (range, 0.03 to 5.9°) and 1.3° (range, 0.3 to 9.6°). Nineteen of the 20 compactors (95%) and 17/20 short stems (85%) were correctly aligned. The proportions of correctly aligned compactors and stems were not significantly different (95% CI, −0.33 to 0.11; Z-test of proportions *p* = .60), and the respective angular deviations were significantly correlated (Spearman ρ = .60, *p* = 0.006). The diaphyseal and metaphyseal filling ratios of the compactors and the stems were all low.

**Conclusions:**

In this series of 20 implants in cadavers, the narrow short humeral stems implanted with compactors for standard-length stems were correctly aligned with the humeral axis. This approach may be a way to achieve both correct frontal alignment and low filling ratios.

## Introduction

Uncemented short stems (length, 50–100 mm) were introduced in the last decade [1–3] in shoulder arthroplasty, with a metaphyseal fixation in press-fit. They are now commonly implanted in current surgical practice [4,5]. Although reported midterm clinical results are good [6–13], some studies report signs of stress shielding [3,7–9,12,14], classically observed in uncemented standard-length (> 100 mm) stems [10,15,16]. Stress shielding occurs because of changes in load distributions according to Wolff’s law, that lead to bone remodeling including tuberosity resorption, cortical thinning, and medial calcar osteolysis. Schnetzke et al. [17] showed that diaphyseal filling ratio (the ratio of the diameters of the stem and the humerus) >0.7 was a risk factor for stress shielding. A high rate of frontal misalignment which is a risk factor for endocortical contact and bone remodeling [18], is reported in between 5 and 47% of cases [19]. Valgus positioning seems to be associated with female gender, older age and a deltoid-sparing approach [19], while varus positioning seems to be associated with lower filling ratios and may result in arthroplasty failure in elderly women [17]. A narrower but misaligned stem carries a higher risk of distal endocortical contact, also associated with stress shielding and bone remodeling [18,20]. The challenge is therefore to implant the thinnest possible short stem without compromising its primary stability, and to align the stem with the humeral axis. During surgery, frontal alignment of the stem depends on the orientation and height of humeral head cut, entry point in the humerus, and the positioning of the compactors during humeral calibration. Peduzzi [9] highlighted the importance of humeral cut height on axis stem deviation: when the cut is too low the remaining neck can lead to valgus positioning while a too high cut can lead to varus positioning of the stem. The frontal orientation of the compactor is under the appreciation of the surgeon, without systematic radiographic use in current practice. Standard-length stems are commonly placed in anatomic alignment compared with short stems [2].

The aim of this cadaver study was to investigate whether correct frontal alignment (an angular deviation of less than ± 5°) [9,21] and optimal filling ratio (<0.7) [21], can be achieved by implanting uncemented short stems using compactors for uncemented standard-length stems. The primary hypothesis was that the standard-length compactors would be aligned with the longitudinal axis of the humerus. The secondary hypothesis was that the short stems would be aligned with the compactors and therefore with the humeral axis, while nevertheless having low filling ratios.

## Materials and methods

### Study design

Twenty humeral stem implantations and fluoroscopic control (described in detail in section “Radiological procedure”) were performed bilaterally on 10 fresh cadavers by one senior shoulder surgeon (SG-7 years experience in shoulder surgery). Another surgeon (PC-10 years experience in shoulder surgery) performed the fluoroscopic controls and kept them blind to avoid any correction in implant positioning with the help of fluoroscopy. The cadavers were obtained from anatomy laboratory of Lyon Medicine School. (six women, four men; mean age at death, 81.3 years; range, 42–96 years). The exclusion criteria were previous shoulder surgery or shoulder fracture sequelae. The cadavers were removed from the cold room twenty-four hours before operations, which were performed at room temperature.

This study was exempted from IRB review in accordance with the exemption criteria laid down by the institutional review board of our hospital (Hospices Civils de Lyon, Lyon, France). The Ethics committee did not ask for any consent. Cadavers were obtained from the Anatomy department of Lyon 1 University, which is a French institution that handles cadavers for research and education purposes. None of the authors had access to patient data identifications.

**Fig 1:**
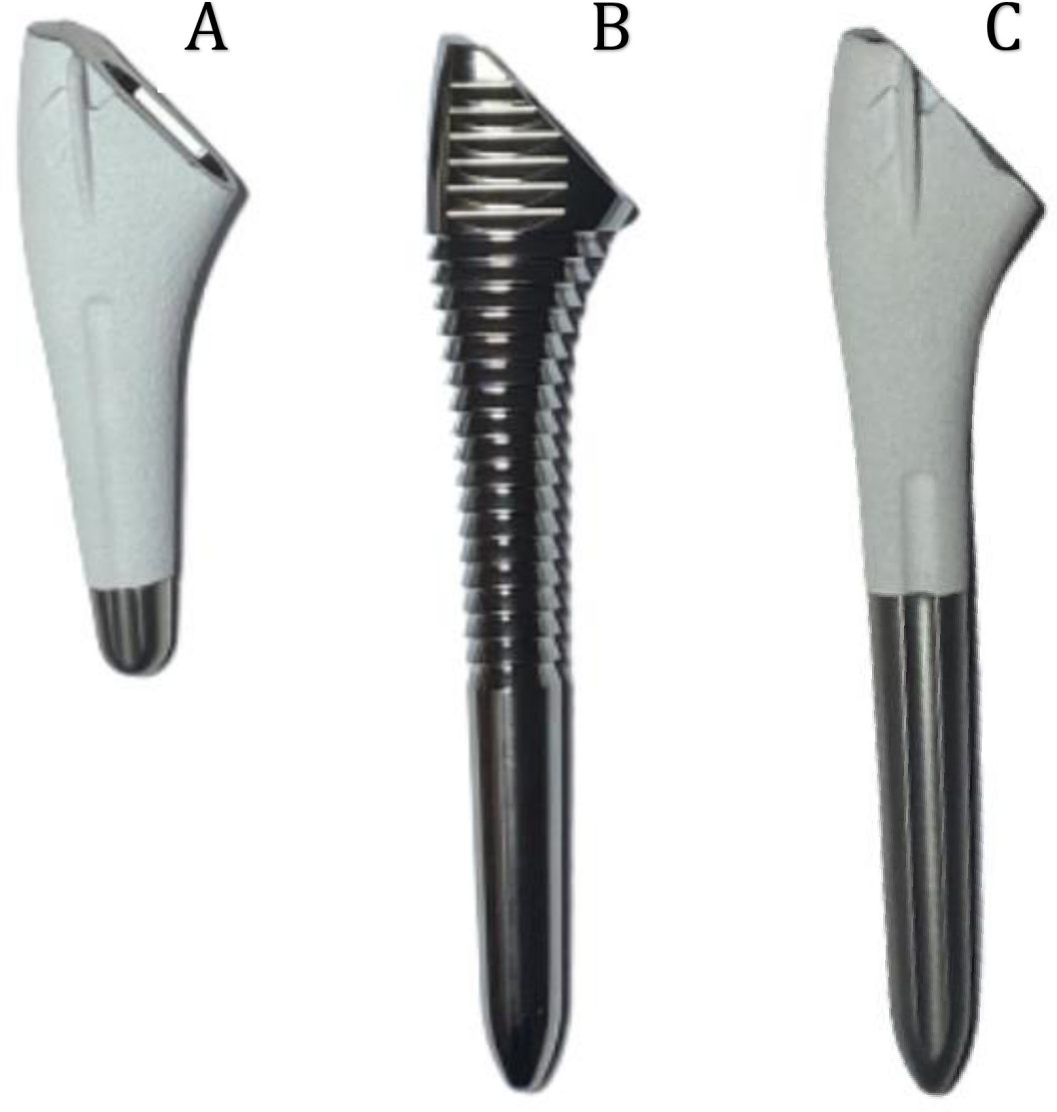
The Evo© short stem (A), Aramis© standard-length compactor (B), and Aramis© standard-lenght Stem (C). The proximal design is similar, the only difference is the length.

The uncemented short stems used in this study were Evo© prostheses (3S Ortho, Lyon, France, Table 1 and Fig 1). The stems are made of titanium, straight, with a low profile, and proximally coated with hydroxyapatite to allow osteo-integration, with two flanges for rotational stability. The system is convertible, with stem inclination of 140° for reverse shoulder arthroplasty. The proximal design is identical to that of Aramis© standard-length stems (3S Ortho, Lyon, France).

**Table 1:** Length of Aramis© compactor and Evo© stems.

Diameter (mm)	7	8,5	10	11,5	13
Aramis**©** compactor length (mm)	112	117	122	127	132
Evo**©** length (mm)	66,52	70,8	75	79,6	83,6

Aramis© standard-length stem instruments were used in all cases. The bone compactors for standard-length stems have the same shape as the corresponding humeral stems, and the same lengths and diameters (Fig 1). The lengths and diameters of the Evo© stem and the Aramis© compactors are reported in Table 1. The proximal and distal diameters of the compactors grow with their size number.

### Shoulder dissection

All cadavers were placed in the beach chair position. A standard operative technique was used without pre-operative planning. After deltopectoral approach, 2 cm of the pectoralis major tendon was released and the subscapularis tendon (if intact) was tenotomized at the anatomical neck of the humerus. The long head of the biceps tendon was also cut if still present. An entry point was made at the top of the humeral head, 1 cm medially and posteriorly from the intertubercular groove. The humeral head was cut using a guide with a 140° neck angle and 10° of retroversion.

After using a canal finder to create a pilot hole in line with the medullary cavity, sounders were used to determine the maximum size of the cavity. The humerus was progressively prepared with a standard-length compactor in 10° retroversion, to preserve the cancellous bone. The aim of the compactors is to create a bed of cancellous bone for definitive stem implantation. Compaction was therefore stopped at the first rotationally stable compactor size, not the maximum size, defined as press-fit. The goals were to implant the narrowest possible stem to minimize the metaphyseal and diaphyseal filling ratios; and to align the stem and the compactor with the diaphyseal axis to avoid cortical contact. Anteroposterior fluoroscopy was performed once the compactor was in place.

The corresponding short stem was then implanted with the same axis and retroversion as the compactor. The rotational stability of the implant was tested to confirm the size match. If the press-fit was found to be loose, the implant size was increased to obtain good stability. Anteroposterior fluoroscopy was performed once more with the same protocol to obtain the same AP view of the humerus as for the compactor.

### Radiologic evaluation

**Fig 2:**
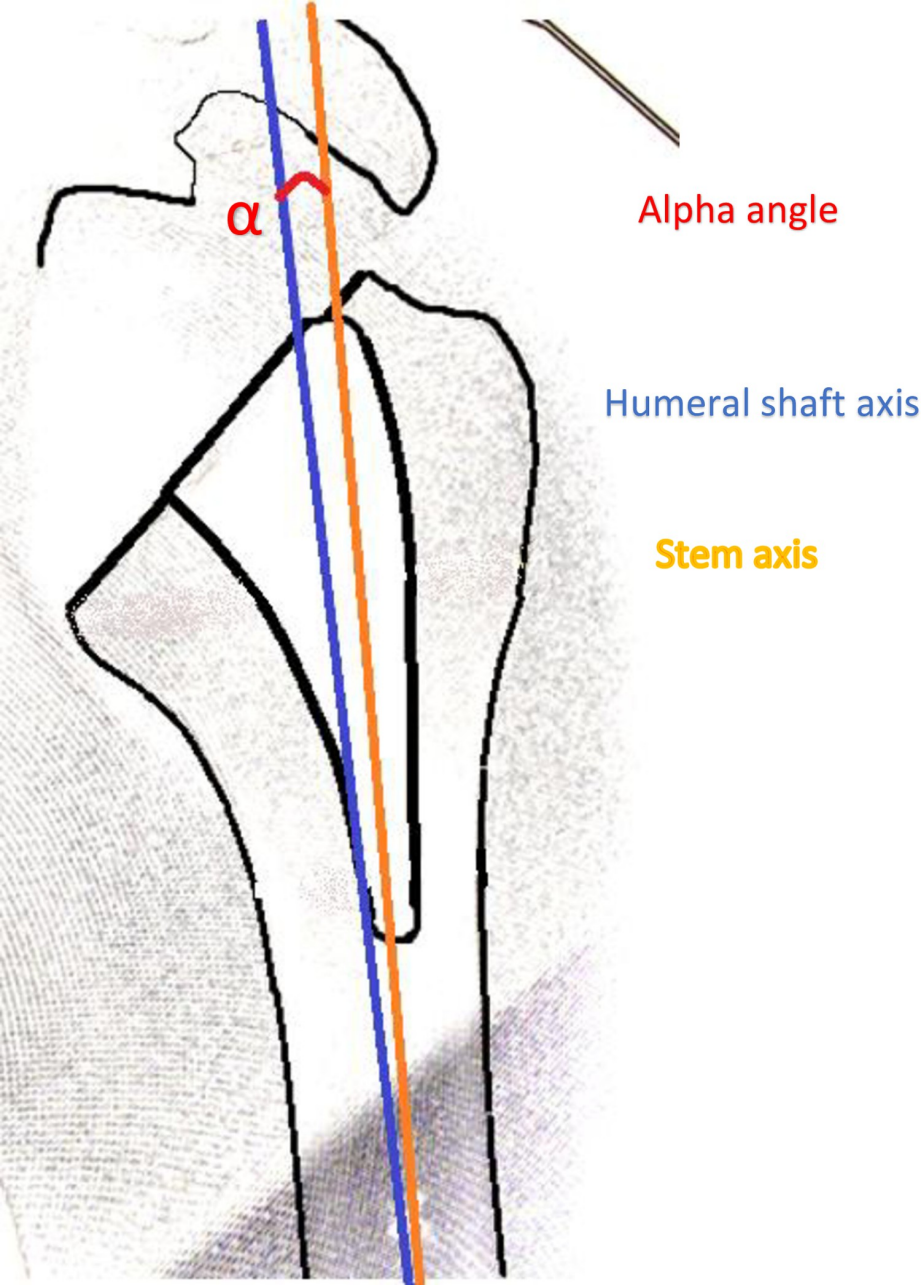
Humeral draw of anteroposterior radiograph. Stem axis deviation obtained by measuring the alpha angle (red circle, 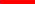) between the axis of the stem (orange line, 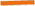) and the axis of the humeral shaft (blue line, 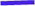).

The same fluoroscopic control protocol was used to observe the standard-length humeral compactors and the short stems: humeral rotation was progressively applied to obtain comparable *anterior-posterior views of the two components*. All measurements were performed with GeoGebra Classique (6.0.574.0, International GeoGebra Institute, Austria). The variables measured were the longitudinal axis of the humerus, the axes of the compactor and of the stem, as described by Peduzzi et al. [9] (Fig 2), using the α angle to compare the latter two with the humeral axis. Neutral alignment was defined according to the literature as a deviation |*α*| < 5° [2]. Valgus was defined as *α* > +5°, and varus *α* < −5°. The correlation between the α angles of the compactors and the stems was calculated.

**Fig 3:**
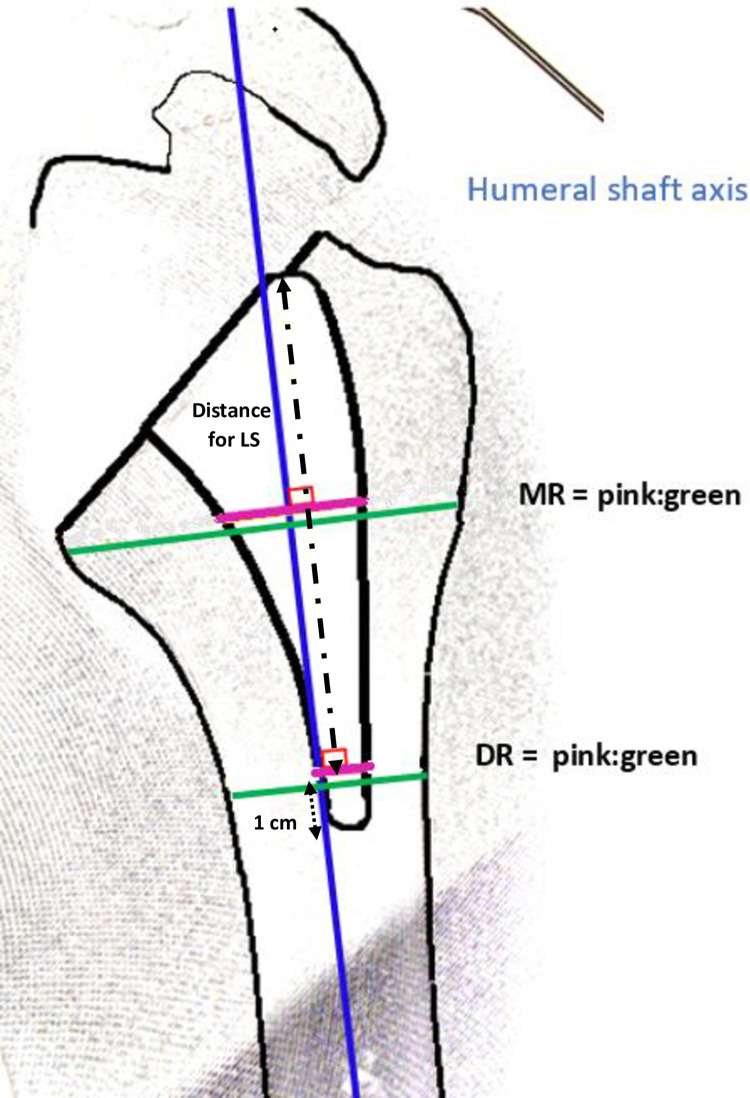
Humeral draw of anteroposterior radiograph. Metaphyseal filling ratio (MR) and diaphyseal filling ratio (DR) obtained by calculating the ratio between the stem diameter (pink line, 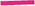) and the diaphysis diameter (green line, 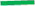). The measured distances are perpendicular to the axis of the humeral shaft. The MR is measured at the level of the cut, and the DR is measured 1 cm over the tip of the short-stem (dotted arrow, 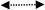), and at that same level for long stem (dashes arrow, 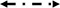).

The metaphyseal and diaphyseal filling ratios were measured as described by the same authors [9] as the ratio of the diameters of the stem or compactor and of the humerus. These were measured perpendicular to the axis of the humeral shaft. The metaphyseal ratio was measured at the level of the cut, and the diaphyseal ratio 1 cm above the tip of the short stem. The metaphyseal and diaphyseal ratios of the compactor were measured at these same levels (Fig 3). Filling ratios > 0.7 were defined as high [21]. Contact between the stem and the cortical bone of the humerus was systematically investigated and reported.

### Statistical analysis

The variables of interest (angular deviations, filling ratios) were summarized as median and range. The normality of the distributions was assessed using Shapiro–Wilk tests. The proportions of misaligned compactors and stems were compared using a two-proportion z-test. Correlations were assessed using Spearman’s rank correlation coefficient. Statistical significance was defined as p < .05. All analyses were performed with R 4.0.5 (R Core Team (2021), www.R-project.org).

## Results

### Stem size

The implanted short stems were size 7 in 8 cases, size 8.5 in 11 cases, and size 10 in one case. For each specimen, the same stem size was implanted for left and right side except in one case (compactor and stem size 8.5 in the right side, and size 10 in the left side, α angle respectively 0.86 and 1.63°).

### Stem alignment

**Fig 4:**
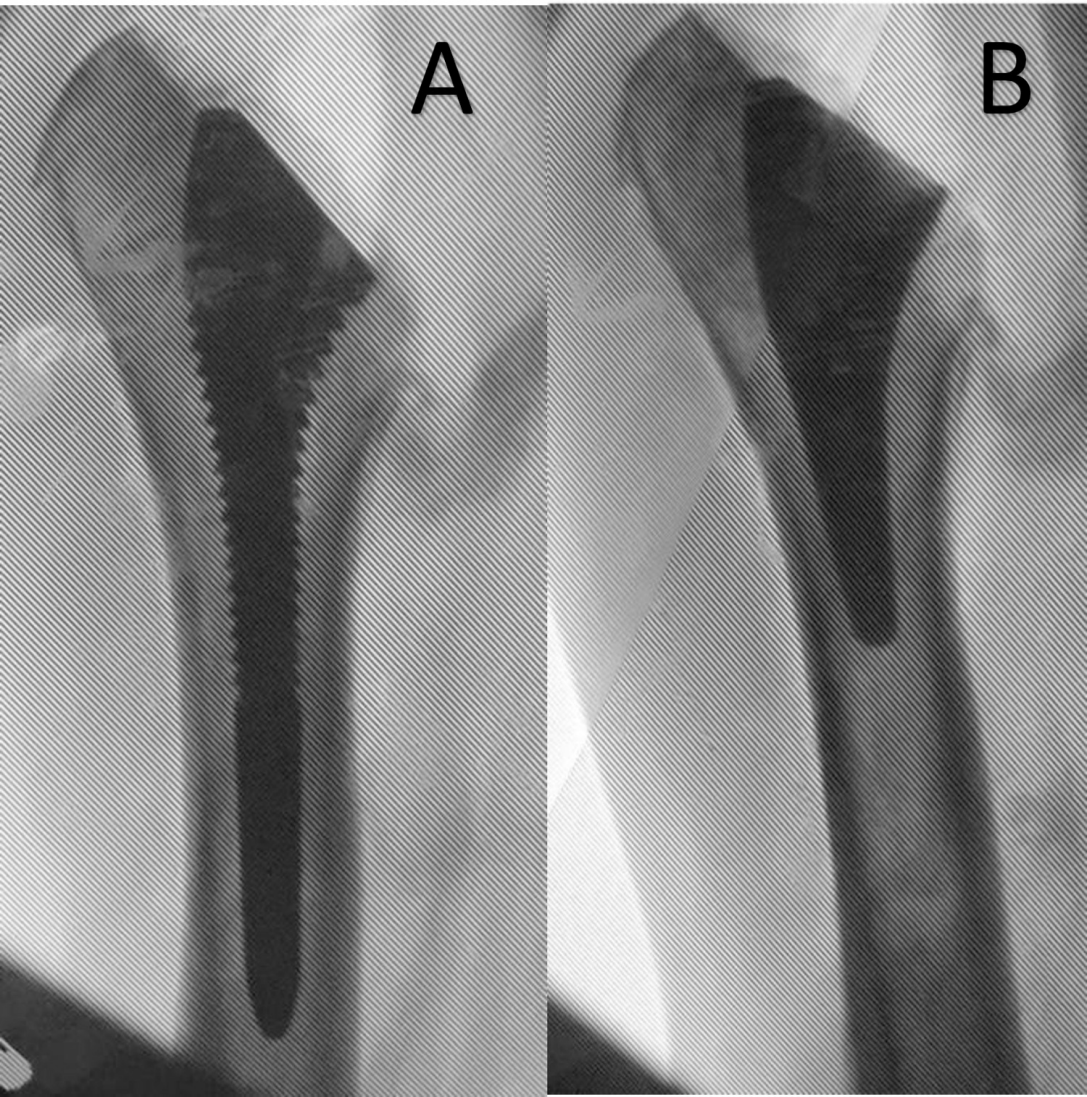
True anteroposterior humeral radiographs showing well aligned implants (size 7), without cortical contact (**A**) trial (alpha angle 0,5°), (**B**) short-stem (alpha angle 0,1°), in the same subject.

**Fig 5:**
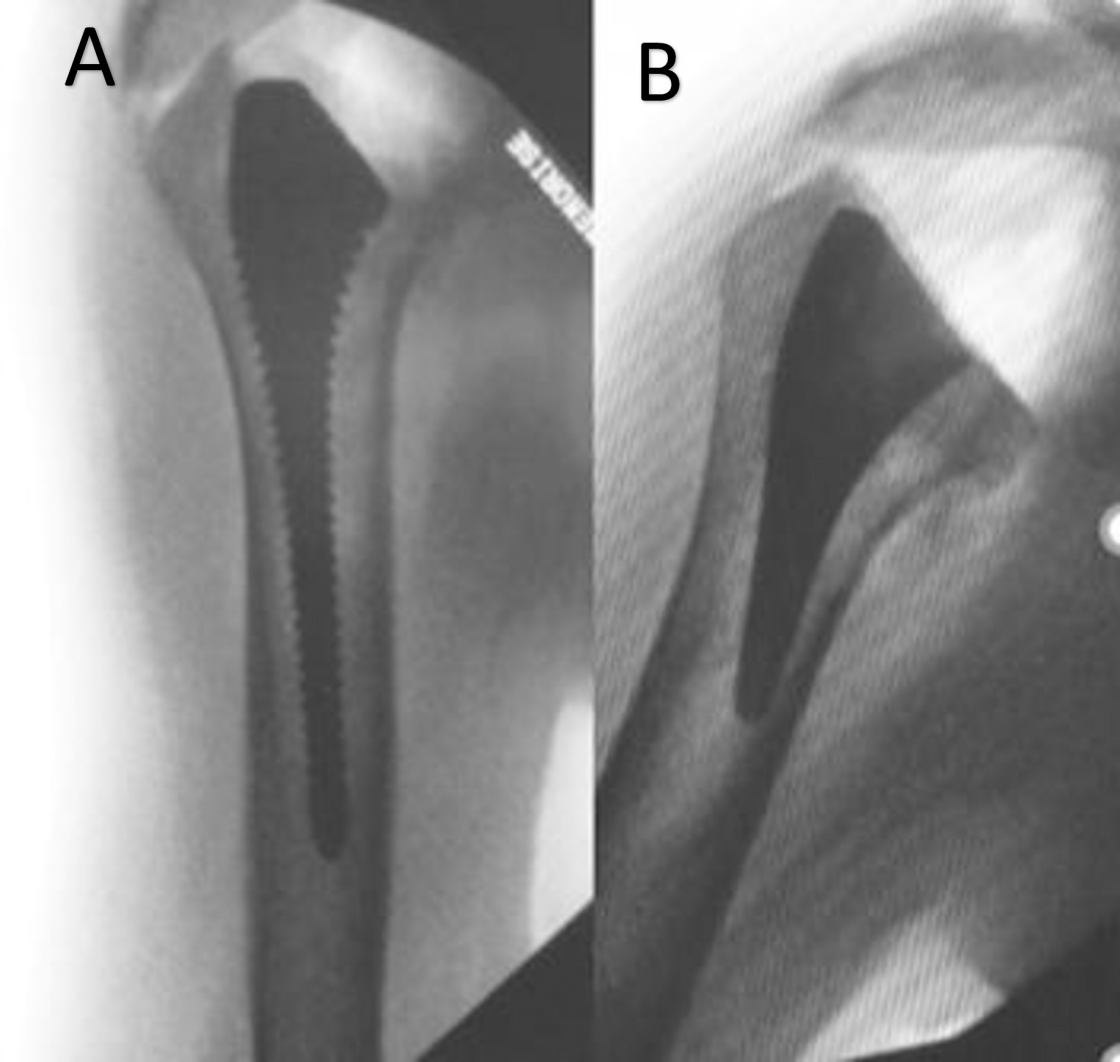
True anteroposterior humeral radiographs showing valgus positioned implants (size 7) (**A**) trial (alpha angle 6,8°) (**B**) short-stem (alpha angle 8,2°) in the same subject.

The mean α angles of the standard-length compactors and the short stems were respectively 1.6° (range, 0.03 to 5.9°) and 1.3° (range, 0.3 to 9.6°; Table 2). Nineteen compactors (95%) and seventeen short stems (85%) were correctly aligned (|*α*| < 5°; Fig 4). The misaligned compactor was in valgus (*α* = +6°), as were the three misaligned stems (*α* = +5.5°, +8.5° and +9.6°, respectively; Fig 5). In each of these three cases, the compactor was also in valgus (*α* = +4°, +4° and +6°, respectively). The degree of misalignment was always less than 10°. All but one compactor (95%) and 17/20 stems (85%) were correctly aligned. The difference in the proportions of correctly aligned stems and compactors was not statistically significant (95% confidence interval on the difference in proportions, −0.33 to 0.11; z-test of proportions *p* = .60). One misaligned stem was size 7, and two were size 8.5, in three separate individuals. In each of these cases, the contralateral stem was the same size, and correctly aligned. The α angles of the compactors and of the stems were significantly correlated (Spearman ρ = 0.60, *p* = .006). No significant difference was found when comparing compactor and stem angular deviations between left and right shoulders (Table 3).

**Table 2:** Patient (cadaver) age and measured implant variables for short humeral stems implanted with standard-length compactors.

	Compactor (n = 20)	Stem (n = 20)	*ρ*	*P*
Age of death	88 (42–98) years			
Angular deviation*	1.6° (0.03–5.9°)	1.3° (0.3–9.6°)	.60	.006^†^
|α| < 5°	19 (95%)	17 (85%)		.60^‡^
Mean difference (95% CI)	−0.02 (−0.04 to 0.001)			.06^§^
Filling ratio				
Metaphyseal	0.42 (0.24–0.65)	0.45 (0.34–0.62)		
< 0.7	20 (100%)	20 (100%)		
Diaphyseal	0.40 (0.29–0.44)	0.41 (0.27–0.50)		
< 0.7	20 (100%)	20 (100%)		

Unless otherwise indicated, data are presented as median (range) or number (%).

*From the longitudinal axis of the humerus.

^†^Spearman rank correlation test.

^‡^z-test for proportions.

^§^paired t-test.

**Table 3:** Compactor and stem angular deviations compared between left and right shoulders.

	Left shoulder (n = 10)	Right shoulder (n = 10)	*ρ*	*P*
Angular deviation*				
Compactor	0.9° (0.4–5.9°)	1.9° (0.03–4.8°)	−0.16	.66^†^
|α| < 5°	9 (90%)	10 (100%)		1.0^‡^
Stem	2.1° (0.3–9.6°)	1.3° (0.5–5.5°)	−0.18	.63^†^
|α| < 5°	8 (80%)	9 (90%)		1.0^‡^

Unless otherwise indicated, data are presented as median (range) or number (%).

*From the longitudinal axis of the humerus.

^†^Spearman rank correlation test.

^‡^z-test for proportions.

### Filling ratios

The metaphyseal and diaphyseal filling ratios of the compactors and stems were all low (< 0.7; Table 2). The mean metaphyseal ratios of the compactors and of the stems were 0.43 and 0.45, respectively, and the corresponding diaphyseal filling ratios were 0.37 and 0.39 (Table 2). Filling ratios were not correlated with angular deviations (Spearman rho = 0.13, p = 0.433; and Spearman rho = 0.05, p = 0.77; between *α* and the metaphyseal and diaphyseal filling ratios, respectively).

### Cortical contact

One instance of cortical contact was observed, for the most misaligned stem. In this case, a size 7 compactor was implanted in valgus with a 4.3° α angle. The size 7 short stem was implanted in valgus position with a 9.6° α angle, that lead to a contact between the tail of the stem and the medial cortex of the humerus.

## Discussion

We evaluated the frontal alignment and filling ratios of short stems implanted after humeral preparation with standard-length compactors. Standard length compactors were correctly aligned with the humeral axis in 95% of the cases, confirming our primary hypothesis. Our secondary hypothesis comparing short stems alignment with the compactors and thus with the humeral axis, while respecting low filling ratios was also confirmed. The main finding of this cadaver study is that standard-length compactors can be used to implant narrow short stems that are correctly aligned with the humeral axis.

The first studies of uncemented short stems reported high levels of bone remodeling due to stress shielding [10]. Raiss et al. [21] found that filling ratios above 0.7 were associated with a seven-fold higher risk of radiographic stress shielding and recommended implants with narrower stems if used without cementing. Although stress shielding may indeed be reduced with thinner stems, the risk of misalignment in varus or valgus is much higher [12,18]. A narrower but misaligned stem carries a higher risk of distal endocortical contact, also associated with stress shielding and bone remodeling [18,20]. Larger-diameter stems with larger filling ratios are less likely to be misaligned but more likely to induce stress shielding. The challenge is therefore to ensure the stem is aligned without increasing the filling ratio to avoid stress shielding.

The standard-length compactors used in this study to insert short stems had the same proximal shape as the equivalent short stems, the only difference being their length. The implanted short stem was in each case the same size as the last used compactor, allowing good press-fit. Oversizing the length of the compactor did not therefore compromise the primary metaphyseal stability of the implanted stems. Denard [2] compared the alignment of short and standard-length stems with a similar proximal design, implanted respectively after humeral preparation with short and standard-length compactors. He demonstrated that standard-length stems were placed in anatomic alignment in 98% of cases, whereas the short stem was placed anatomically in 86% cases.

The strong correlation found here between the orientations of the compactors and the stems suggests that short stems implanted in this way remain aligned with the compactor. In previous studies of short-stem positioning, the compactors used were of the same shape and length as the final humeral implant [10,19]. In a series of 159 reverse shoulder arthroplasties, Lädermann et al. [19] observed stem misalignment exceeding 5° in 47% of shoulders, 9% in varus, and 38% in valgus. Valgus positioning is the most frequently reported problem [9,19,22,23], with maximum deviations of 11.5–32°, while the misalignments in our study were all less than 10°. The proportion of correctly aligned (|*α*| < 5°) short stems in our cadaveric series (85%) is in keeping with the misalignment rate reported by Raiss et al. [21] (16%) in their radiographic study of short stems. Our results therefore suggest that using standard-length compactors to implant short stems carries a low risk of misalignment.

The aims of humeral preparation are threefold; first, preserve cancellous bone to avoid cortical contact; second, implant the thinnest possible stem to avoid stress-shielding; and third, obtain good metaphyseal press-fit fixation. With short compactors, the implant may have to be oversized to ensure good stem alignment and sufficient proximal primary fixation. Press-fit implantation with a high filling ratio [13,21,22,24,25] has been identified as a risk factor for bone adaptation, particularly when the diaphyseal filling ratio is above 0.7 [21]. Stopping compaction at the first size with rotational stability avoids having to then oversize the stem [1,9,21] to achieve good press-fit and primary stability. In the present study, the implanted stem was size 7 or 8.5 in 90% of cases. A size 10 stem was only implanted in one case (5%), a male cadaver with a low diaphyseal filling ratio (0.4). The filling ratios in our series are lower than previously reported [9,19,21] and were not correlated with stem alignment. The diaphyseal filling ratio was at most 0.50 and 0.38 on average, well below Raiss et al.’s [21] recommended upper limit of 0.7.

Maintaining a lateral entry point in the humerus while broaching is important to avoid varus positioning of the final implant [26]. In our series, three stems were misaligned in valgus, and all the stems reproduced the slight valgus alignment of the compactor. Peduzzi et al. [9] have described how a cautious humeral head cut at the correct height is essential for good stem positioning, and that cutting too low leads to valgus deviation, while cutting too high leads to varus deviation. When analyzing the three stems implanted in valgus we noticed that in each case, the humeral cut was high, with large humeral osteophyte and massive cuff tear. The misalignment may therefore have been the result of an unsuitable entry point, too lateral into the humerus. In cases of massive cuff tear or severe arthropathy, joint modifications can make bone landmarks and the correct entry point (in line with the humeral shaft) difficult to identify. Pre-operative planning and careful optimization of the entry point into the humerus are therefore required to achieve good frontal alignment [1].

The limitations of our study include its small size and the absence of any clinical assessment or radiographic follow-up due to its cadaveric nature. Secondary varus positioning [17], can therefore not be ruled out, and only one type of prosthesis was used without pre-operative planning. Bone quality may also not have been representative because the subjects were older at death than patients generally are when undergoing shoulder arthroplasty. Bone density can also be modified by low humeral canal temperature, and rotational stability can be subjective while compacting metaphyseal cancellous bone. The absence of muscular tonus in the cadavers may have made humeral exposure and stem implantation through a deltopectoral approach easier than reported in the literature [19]. Finally the lack of control group to compare short stem implantation following metaphyseal preparation with short compactors can be discussed. Further studies after in vivo implantations with clinical follow-up are required to confirm these results in practice and evaluated long-term outcomes, notably in terms of stress-shielding induced bone remodeling.

The strengths of this study are the standardized and reproducible nature of the procedure considered, which corresponds to daily practice, the reproducible radiographic measurements and simple, noninvasive, operative technique with standard compactors, and without necessity of supplementary instrumentation.

## Conclusion

Standard length compactors were correctly aligned with the humeral axis in 95% of the cases, confirming our primary hypothesis. Our secondary hypothesis comparing short stems alignment with the compactors and thus with the humeral axis, while respecting low filling ratios was also confirmed. The results of this study performed in a cadaver model suggest that using standard-length compactors to implant short humeral stems is a good option to implant thin stems with a low risk of misalignment, a low risk of cortical contact, and low filling ratios, as recommended.

## Supporting information

S1 Data (XLSX)
